# Defining the Efficacy of Meningococcal B Vaccines Against Gonococcal Acquisition: The Current Landscape

**DOI:** 10.1002/jia2.70153

**Published:** 2026-07-24

**Authors:** Kate L. Seib, Andrew E. Grulich

**Affiliations:** ^1^ Institute for Biomedicine and Glycomics Griffith University Gold Coast Queensland Australia; ^2^ The Kirby Institute University of New South Wales Sydney New South Wales Australia

**Keywords:** 4CMenB, gonorrhoea, meningococcal B vaccine, *Neisseria gonorrhoeae*, vaccine

## Abstract

**Introduction:**

Gonorrhoea is a major global sexually transmitted infection, with rising incidence and increasing antimicrobial resistance threatening current control strategies. If untreated, *Neisseria gonorrhoeae* can lead to severe reproductive health sequelae. Gonorrhoea is also linked to increased HIV acquisition and transmission. There is currently no vaccine licensed to prevent gonorrhoea. However, observational evidence suggests that outer membrane vesicle–based serogroup B meningococcal vaccines, including the four‐component meningococcal B (4CMenB) vaccine, confer partial cross‐protection against gonorrhoea. We discuss observational studies and randomized controlled trials (RCTs) focused on defining the efficacy of 4CMenB against gonorrhoea.

**Discussion:**

Observational studies from multiple settings have reported an association between receipt of 4CMenB vaccine and reduced gonorrhoea risk, with a meta‐analysis estimating a 38% reduction in risk. *Neisseria meningitidis* and *N. gonorrhoeae* are closely related bacteria that share numerous antigens, making cross‐protection biologically plausible. Based on observational data, 4CMenB immunization programmes have been implemented in two countries with the aim of preventing gonorrhoea. However, three RCTs have recently shown that 4CMenB is not effective in preventing gonorrhoea in gay, bisexual and other men at high risk of acquisition. Several RCTs are ongoing, looking at efficacy in different populations, including women and people at lower risk of acquisition. The different outcomes between the observational studies and RCTs may be due to a range of known and unknown confounding factors, and differences in the populations considered in the different studies.

**Conclusions:**

Current evidence from RCTs does not support the use of 4CMenB to prevent gonorrhoea in gay and bisexual men at high risk of acquisition. Results from ongoing RCTs will be critical to determine whether vaccine efficacy varies by population or epidemiological context and to inform future gonorrhoea vaccine policy and development.

## 1 Introduction

Gonorrhoea, caused by *Neisseria gonorrhoeae*, remains a global public health priority, with more than 82 million new cases estimated annually and rising resistance to available antibiotics [1]. Control strategies rely on screening and treatment, yet incidence continues to increase in many regions, particularly among key populations including gay, bisexual and other men who have sex with men (GBMSM), sex workers, transgender people, people living with HIV, racial and ethnic minorities, Indigenous populations, those with limited access to sexual health services, and adolescents and young adults [2, 3]. If untreated, *N. gonorrhoeae* can lead to severe reproductive health sequelae, including pelvic inflammatory disease, chronic pelvic pain, ectopic pregnancy and tubal infertility in women, epididymitis in men and less commonly disseminated disease [4]. Gonorrhoea is also associated with an increased risk of HIV [5].

Vaccine development for sexually transmitted infections (STIs) remains challenging (see “A decade of the global STI vaccine roadmap” in this supplement [6]). For gonorrhoea, these challenges reflect antigenic variability and the lack of durable protective immunity after acquisition, with no known immune correlates of protection. Vaccine candidates evaluated in human clinical trials to date have not demonstrated efficacy [7], and the NgG vaccine, based on genetically detoxified *N. gonorrhoeae* outer membrane vesicles (OMVs), will not progress beyond Phase II [8, 9]. A major shift in the field resulted from observational studies reporting that OMV–based meningococcal vaccines, developed to prevent invasive disease caused by serogroup B *Neisseria meningitidis*, are associated with a reduced risk of gonorrhoea, as reviewed in [10, 11, 12]. Ecological studies have reported that vaccination campaigns implemented to control epidemics using MeNZB in New Zealand [13], VA‐MENGOC‐BC in Cuba [14] and MenBvac in Norway [15] were associated with reduced rates of gonorrhoea. Subsequent research has focused on the four‐component meningococcal B (4CMenB) vaccine, which contains OMVs equivalent to the MeNZB vaccine and three recombinant proteins (fHbp fused to genome‐derived *Neisseria* antigen GNA2091, NadA and NHBA fused to GNA1030) [16]. Several case−control and cohort studies have associated 4CMenB with a reduced risk of gonorrhoea [17, 18, 19, 20, 21, 22, 23, 24, 25], and a recent meta‐analysis estimated a 38% reduction in risk of gonorrhoea following two doses of 4CMenB (95% confidence interval [CI] 28%−48%) [10]. On the basis of these findings, two 4CMenB immunization programmes for gonorrhoea prevention were initiated in 2025 in higher‐risk populations: one in Galicia, Spain targeting adults aged 18–65 years at high risk of gonorrhoea [26], and another in the United Kingdom targeting GBMSM at high risk [11]. These observational data are biologically supported by the close genetic relationship between *N. meningitidis* and *N. gonorrhoeae* and the ability of 4CMenB to induce cross‐reactive immune responses. However, since 2024, three randomized controlled trials (RCTs) of 4CMenB conducted among men who have sex with men (MSM) at high risk reported no reduction in gonorrhoea risk [27, 28, 29, 30, 31]. Several RCTs are underway alongside ongoing observational studies.

In this commentary, we discuss the current landscape of observational studies and RCTs evaluating 4CMenB‑mediated protection against gonorrhoea, how differences in study design and population may explain divergent results, and implications for vaccine policy and future research.

## 2 Discussion

### 2.1 Observational Studies Investigating Cross‐Protection of 4CMenB Against Gonorrhoea

Case–control and retrospective cohort studies from Australia, the USA, Canada and Italy have consistently reported an association between 4CMenB and a reduction in gonorrhoea risk ranging from 25% to 45% across settings, age groups and study designs, with a greater reduction in risk associated with two doses (see Table 1).

**TABLE 1:** Summary of observational studies, randomized controlled trials and controlled human infection models evaluating 4CMenB and gonorrhoea outcomes.

Study type/name [Ref]	Location	Population/eligibility criteria	Control/comparator	Sample size/outcomes	Measure of association
**Observational, case−control studies**					
Retrospective case−control [25]	New York City and Philadelphia, USA	Individuals aged 16–23 years, with gonorrhoea and chlamydia captured in surveillance records	Chlamydia‐only cases	18,099 gonorrhoea only cases, 24,731 gonorrhoea and chlamydia cases, 124,876 chlamydia only cases	Unadjusted prevalence ratio = 0.64 (95% CI 0.51−0.79); adjusted prevalence ratio = 0.60 (95% CI, 0.47−0.77)^a^
Retrospective case−control [24]	South Australia	School immunization programme targeting ages 15–20 years	Chlamydia cases	1613 gonorrhoea case patients and 7609 chlamydia controls	Adjusted odds ratio = 0.61 (95% CI, 0.54−0.69)^b^
Nested case−control study [17]	Northern California, USA	Individuals aged 15–30 years, with gonorrhoea and chlamydia captured in the electronic health records of Kaiser Permanente Northern California	Chlamydia cases	10,638 people with gonorrhoea only; 53,914 people with chlamydia only	Prevalence ratio with limited adjustment = 0.77 (95% CI, 0.64−0.99); with full adjustment 0.99 (95% CI, 0.79−1.25) ^c^
Unmatched case−control [20]	Milan, Italy	MSM living with HIV under clinic care with a previous STI diagnosis	Other STIs	103 gonorrhoea cases, 948 controls with syphilis, chlamydia or anal human papillomavirus	Unadjusted odds ratio = 0.58 (95% CI, −36 to 94); adjusted odds ratio = 0.56 (95% CI, 0.35−0.91)^d^
B part of it NT, case−control [32]	Northern Territory, Australia	Individuals aged 14–19 years in the Northern Territory (NT)	Chlamydia cases	*N* = ∼7100	Ongoing
**Observational, cohort studies**					
Retrospective cohort [23]	Oregon, USA	University students aged 18–29 years following mass vaccination campaigns prompted by meningococcal serogroup B (MenB) outbreaks	MenB‐FHbp vaccine	15,760 4CMenB recipients, 15,212 MenB‐FHbp recipients	47% reduction in the proportion of people diagnosed with gonorrhoea (95% CI, 13%−68%)
Retrospective matched cohort [21]	Southern California, USA	Individuals aged 15–30 years, with data captured from electronic health records at 15 hospitals and associated medical offices	MenACWY vaccine	6641 4CMenB recipients, 26,471 MenACWY recipients	Unadjusted hazard ratio = 0.38 (95% CI, 0.26−0.56); adjusted hazard ratio = 0.54 (95% CI, 0.34−0.86)^e^
**Randomized controlled trials**					
DOXYVAC—open‐label [29]	Paris, France	MSM aged ≥18 years; HIV‐negative; using PrEP; documented bacterial STI within the previous 12 months; vaccination allowed in the presence of a current case of gonorrhoea	No vaccine	*N* = 544 (modified intention‐to‐treat population) 58.3 versus 77.1 cases per 100 person years in 4CMenB versus control group, respectively	Adjusted hazard ratio = 0.78 (95% CI, 0.60−1.01; *p* = 0.061) for first‐episode gonorrhoea
MenGO—open‐label [28, 31]	Gold Coast, Australia	MSM aged 18–50 years; receiving PrEP or confirmed gonorrhoea within the previous 3 months; vaccination allowed in the presence of a current case of gonorrhoea	No vaccine	*N* = 130 (per‐protocol population) 27.1 versus 34.8 cases per 100 person years in 4CMenB versus control group, respectively	Incidence rate ratio = 0.78 (95% CI, 0.40−1.51; *p* = 0.457) for all‐episode gonorrhoea
GoGoVax—double‐blind, placebo‐controlled [27, 30]	Sydney, Melbourne and Gold Coast, Australia	MSM aged 18–50 years; living with HIV or HIV‐negative and using PrEP; history of gonorrhoea or infectious syphilis within the previous 18 months; vaccination allowed in the presence of a current case of gonorrhoea	Saline placebo	*N* = 587 (per‐protocol population) 48.1 versus 47.8 cases per 100 person years in 4CMenB versus placebo group, respectively	Incidence rate ratio = 1.01 (95% CI, 0.80−1.25; *p* = 0.97) for first‐episode gonorrhoea
BIYELA—observer‐blind, placebo‐controlled [33]	Cape Town, Johannesburg and Pietermaritzburg, South Africa	Cisgender women aged 18–45 years; sexually active in the past 3 months; PrEP use, history of STIs or ≥2 partners in the past 12 months; negative gonorrhoea and chlamydia test within 14 days prior to randomization	Saline placebo	*N* = ∼1100	Ongoing
MAGI—observer‐blind, placebo‐controlled [34]	USA, Malawi, Thailand	Men and women aged 18–50 years; disproportionately vulnerable to gonorrhoea acquisition (risk criteria not specified); negative gonorrhoea and chlamydia test within 14 days prior to randomization	Saline placebo	*N* = ∼2200	Ongoing
Double‐blind placebo‐controlled [35]	Hong Kong	MSM aged 18–50 years; condomless sex with >1 man in the last 6 months; history of STIs; meet PrEP‐eligible criteria; negative gonorrhoea test at recruitment	Saline placebo	*N* = ∼150	Ongoing
**Controlled human challenge model**					
Male urethral gonococcal infection model [36]	North Carolina, USA	Men aged 18–35 years; general good health; exclusion includes prior confirmed invasive meningococcal disease	Seasonal influenza and tetanus/diphtheria vaccine	*N* = ∼140	Ongoing

Abbreviations: 4CMenB, four component meningococcal serogroup B vaccine; CI, confidence interval; HIV, human immunodeficiency virus; MenACWY, combined meningococcal serogroup A, C, W and Y vaccine; MenB‐FHbp, meningococcal serogroup B (MenB) factor H binding protein (fHbp) vaccine; MSM, men who have sex with men; PrEP, pre‐exposure prophylaxis for HIV prevention; STI, sexually transmitted infection.

^a^Adjusted for race/ethnicity, gender and jurisdiction.

^b^Adjusted for sex at birth and socio‐economic status.

^c^Limited adjustment included race/ethnicity only. Expanded adjustment included all variables significant in bivariate analysis (race/ethnicity, gender, insurance status, neighbourhood deprivation index, HIV status).

^d^Adjusted for age, hepatitis coinfection, CD8+ and CD4+ count, years of antiretroviral therapy and HIV‐RNA level.

^e^Adjusted for ethnicity, number of outpatient visits, HIV, PrEP use, STI diagnoses and mental health visits in year prior to vaccination.

In South Australia, a publicly funded 4CMenB programme was introduced for children in 2018 and for adolescents and young adults in 2019. Using a case–control method with age‐matched individuals with chlamydia as controls, 5 years after the programme introduction, based on the calculated odds ratio (OR), estimated vaccine effectiveness (1‐OR) for two‐dose vaccination against gonorrhoea in adolescents was 39.1% (95% CI 31.3%−46.0%) [24]. The association waned over time, with lower estimates in those >5 years compared to within 5 years since vaccination (−6.3% [95% CI −44.5% to 21.8%] vs. 41.8% [34.0%−48.7%]). An estimated vaccine effectiveness of 27.0% (adjusted hazard ratio [aHR] 0.730 [95% CI 0.540−0.988]) was seen for subsequent *N. gonorrhoeae* reacquisition when comparing fully vaccinated with unvaccinated case patients [24]. In an earlier evaluation, vaccine effectiveness over 4 years of follow‐up was 48.7% (95% CI 36.9%−58.2%) in females and 38.0% (95% CI 22.0%−50.7%) in males [37].

In New York City and Philadelphia, using a case−control study based on STI surveillance data linked to immunization records from 2016 to 2018, two 4CMenB doses were estimated to be 40% (95% CI 23%−53%) effective, and one dose 26% (95% CI 12%−37%) effective against gonorrhoea in 16‐ to 23‐year‐olds [25]. In Northern California, using a nested case−control study based on gonorrhoea and chlamydia cases in electronic health records of Kaiser Permanente Hospitals and associated medical offices, 4CMenB was estimated to be 23% protective against gonorrhoeae, with a prevalence ratio after adjustment for race/ethnicity of 0.77 (95% CI 0.64−0.99). However, after including adjustment for gender, insurance status, neighbourhood deprivation index and HIV status, the association was no longer seen (0.99 [95% CI 0.79−1.25]) [17].

Two retrospective cohort studies in the USA compared gonorrhoea incidence after 4CMenB vaccination with incidence after non‐OMV‐based meningococcal vaccines that are not predicted to protect against gonorrhoeae. In Southern California, there was a 46% lower incidence rate of gonorrhoea in 15‐ to 30‐year‐olds, but not chlamydia, among 4CMenB recipients compared with matched counterparts who had received meningococcal MenACWY vaccine (HR 0.54; 95% CI 0.34−0.86) [21]. In Oregon, gonorrhoea incidence was 47% (95% CI 13%−68%) lower in 18‐ to 29‐year‐olds who received 4CMenB compared to those who received the MenB‐FHbp vaccine [23]. In an Italian clinic‐based case−control study, in MSM living with HIV and with a history of gonorrhoea, syphilis, chlamydia or anal human papillomavirus, receiving two doses of 4CMenB was associated with a 44% (95% CI 9%−65%) reduced risk in adjusted analyses [20].

Although the estimated efficacy of 4CMenB from observational studies is modest, and assuming it is a causal relationship, mathematical modelling has indicated that the population‐level impact could be substantial when implemented widely and/or targeted to groups at highest risk of acquisition [38]. Cost‐effectiveness modelling indicated that vaccination according to risk would likely be cost‐saving in MSM in the UK [39]. Based on observational studies and health economics modelling, a 4CMenB immunization programme was implemented in the UK in 2025 in MSM at high risk of gonorrhoea [40].

### 2.2 Biological Rationale for Cross‑Protection

The biological rationale for cross‐protection by OMV‐based serogroup B meningococcal vaccines against gonorrhoea is supported by the close genetic relationship between *N. meningitidis* and *N. gonorrhoeae*. The OMV component of 4CMenB contains several antigens that have homologues in gonorrhoea [41, 42], while NHBA is the only recombinant protein that is surface expressed on *N. gonorrhoeae* and able to be recognized by vaccine‐induced antibodies [41]. Other non‐OMV meningococcal vaccines are not predicted to protect against gonorrhoea due to a lack of common antigens. MenB‐FHbp (rLP2086, Trumenba, Pfizer) contains two fHbp subvariants [43]; although fHbp is present in *N. gonorrhoeae*, it is not surface exposed [44]. Meningococcal serogroups ACWY vaccines target the capsule polysaccharide, which *N. gonorrhoeae* lacks. Accordingly, MenB‐FHbp [23] and MenACWY [21] vaccines have been used as controls in observational studies.

In humans and animal models, 4CMenB induces cross‐reactive serum IgG and mucosal IgA/IgG that recognize *N. gonorrhoeae* proteins and lipooligosaccharide [41, 45, 46, 47, 48, 49]. Serum bactericidal activity is a correlate of protection for meningococcal vaccines [50], but no correlates of protection are defined for gonorrhoea. However, in a female mouse model, 4CMenB increases serum bactericidal titres, reduces bacterial load, accelerates clearance in a subset of mice and induces a multi‐faceted cellular immune response [45, 51]. In humans, 4CMenB vaccination also results in increased serum bactericidal activity titres [31, 47, 48].

### 2.3 RCTs of 4CMenB Efficacy Against Gonorrhoea

Several RCTs have been completed or are underway in France, Australia, the USA, South Africa and Hong Kong to directly evaluate 4CMenB efficacy against gonorrhoea. All RCTs use a two‐dose 4CMenB schedule (1−3 months apart) and exclude prior 4CMenB vaccination, but vary in design, participant sex and gonorrhoea history, baseline incidence, and future acquisition risk (see Table 1).

To date, three RCTs have reported no protective effect of 4CMenB against gonorrhoea in MSM at high risk of acquisition [29, 30, 31]. These trials were conducted in settings with very high gonorrhoea incidence (∼31–67 cases per 100 person years), allowed vaccination in the presence of current gonorrhoea, and included frequent screening for asymptomatic and extragenital gonorrhoea. ANRS 174 DOXYVAC was a multicentre, open‐label, RCT in MSM aged ≥18 years in France who were HIV‐negative, using HIV pre‐exposure prophylaxis and had a documented bacterial STI within the previous 12 months [29]. The adjusted hazard ratio (aHR) for first‐episode gonorrhoea was 0.78 (95% CI 0.60–1.01; *p* = 0.061), giving a vaccine efficacy of 22% (95% CI −1% to 40%). For cumulative incidence, the aHR was 0.84 (95% CI 0.67–1.07). GoGoVax was a multicentre, double‐blind, placebo‐controlled RCT in MSM aged 18–50 years in Australia, either living with HIV or HIV‐negative and using pre‐exposure prophylaxis (PrEP), with a history of gonorrhoea or infectious syphilis in the previous 18 months [27, 52]. The incidence rate ratio (IRR) of first‐episode gonorrhoea was 1.01 (95% CI 0.80–1.25; *p* = 0.97), giving a vaccine efficacy of −0.5% (95% CI −26.16% to 19.93%). The IRR for all‐episode gonorrhoea incidence was 0.98 (95% CI 0.81–1.20) [30]. MenGO was a single‐site, open‐label, RCT in MSM aged 18−50 years in Australia receiving HIV pre‐exposure prophylaxis or with confirmed gonorrhoea within the previous 3 months [28]. The IRR for all‐episode gonorrhoea was 0.78 (95% CI 0.40–1.51; *p* = 0.457) [31].

In the MenGO trial, bactericidal antibody titres waned over time and were lower in participants with gonorrhoea at the time of vaccination [31]. *N. gonorrhoeae* uses immune evasion and modulation strategies that hinder long‐lasting immunity [53, 54], and the impact of prior or current infection on vaccine‐induced immune responses is unknown but may reduce potential protection. For example, acquisition induces antibodies to the Rmp protein, which may block effective immune responses to reacquisition or vaccination [55, 56]. Ongoing studies from larger trials that include participants with and without past gonorrhoea, or with active gonorrhoea at the time of vaccination, are needed to confirm these findings.

The outcomes of ongoing RCTs are expected in the next 2 years. BIYELA (Bexsero Immunisation in Young Women in Africa) is a multi‐site, observer‐blind, placebo‐controlled RCT recruiting cisgender women aged 18–45 years at five South African sites who have been sexually active in the past 3 months, and report PrEP use, a history of STIs, or two or more partners in the past 12 months [33]. MAGI (Meningococcal Antigen–Gonococcal Infection) is a multi‐site, observer‐blind, placebo‐controlled RCT in men and women aged 18–50 years in the USA, Thailand and Malawi who are disproportionately vulnerable to gonorrhoea, although specific risk factors are not listed [34]. A study in Hong Kong is a single‐site, double‐blind, randomized placebo‐controlled trial in men aged ≥18 who have had condomless sex with more than one man within the last 6 months, have a history of STI diagnosis, report an inclination to have condomless sex and meet other PrEP‐eligible criteria [35]. Participants in these three studies must have tested negative for gonorrhoea at the time of, or 14 days prior to, enrolment.

A controlled human infection model is being used to evaluate 4CMenB efficacy against urethral gonorrhoea in a single‐site, double‐blind RCT in healthy males 18–36 years old in North Carolina [36, 57]. Controlled human infection models enable assessment of vaccine efficacy under defined exposure conditions, including effects on acquisition, bacterial load and clearance.

### 2.4 The Limitations of Observational Evidence

The absence of demonstrable efficacy in RCTs to date, despite consistent signals from observational studies, highlights the limitations of observational evidence. It has long been recognized that observational studies may report associations between pharmaceuticals and disease that are not causal but are rather due to confounding [58]. There are multiple examples where evidence from observational studies has been later contradicted by RCT results, including in the STI field. Consistent observational data demonstrated that genital herpes is associated with increased risk of HIV, and thus herpesvirus suppression was an attractive HIV prevention candidate. However, three subsequent RCTs showed that effective herpesvirus suppressive therapy did not reduce HIV acquisition or transmission [59, 60, 61]. It is possible that the association between meningococcal B vaccination and gonorrhoea reported in observational studies reflects residual confounding rather than a causal protective effect. Most of the observational evidence on the association between meningococcal B vaccination and gonorrhoea comes from case−control studies where the vaccination history of people with gonorrhoea is compared to that of people with chlamydia [18, 20, 24, 25, 37]. It is plausible that vaccination might be less common in people with gonorrhoea than in people with chlamydia for reasons other than a causal protective effect. For example, there is some evidence that people with gonorrhoea are more socio‐economically deprived than people with chlamydia [37], so there may be financial reasons for lower vaccination rates. Observational data have also been reported from cohort studies, comparing gonorrhoea incidence between recipients of meningococcal B vaccines and those receiving other meningococcal vaccines [21, 23]. However, as the medical indications for receiving different vaccines often differ, these two groups may differ in important characteristics which may influence gonorrhoea risk. While some demographic and other risk differences can be controlled for in observational studies using statistical methods, adjustment for confounders in the studies included here varied markedly. It is likely that residual confounding remains in the adjusted estimates, reflecting differences in determinants of vaccination (e.g. socio‐economic status, healthcare engagement, vaccine access), sexual network structure and STI screening frequency. In at least one well‐conducted large retrospective cohort study in Northern California, 4CMenB vaccination was associated with reduced gonorrhoea risk in a model with limited control for cofounding, but not after full adjustment for a range of confounders [17]. In contrast to observational studies, a well‐conducted RCT reliably ensures the comparison groups are balanced for all known and unknown confounders.

## 3 Conclusions

Observational studies consistently demonstrate an association of 4CMenB with reduced gonorrhoea risk, and the potential causal nature of this association is supported by biological rationale and immunological evidence. However, RCT data to date do not support a protective effect in MSM at high risk of gonorrhoea acquisition. Taken together, these findings indicate that current evidence does not support implementation of 4CMenB for gonorrhoea prevention in populations similar to those enrolled in completed RCTs, and the UK may need to reconsider their recommendations. However, evidence remains insufficient to support or refute its use in other contexts.

The absence of demonstrated efficacy in recent trials raises questions regarding the feasibility of OMV‐based approaches for gonococcal prevention. Although gonococcal‐based OMV vaccines, such as NgG, offer greater antigenic specificity and have been modified to limit immune interference, they may still include immunodominant but non‐protective antigens or elicit blocking antibodies. Nevertheless, effective vaccination may still be achievable if antigens that induce functional immune responses capable of bacterial killing and/or blocking colonization are delivered using appropriate platforms and adjuvants. Several candidates are progressing through the pipeline, including OMV‐based vaccines employing different strains or adjuvant strategies, as well as approaches targeting conserved surface proteins and lipooligosaccharide structures [7].

Vaccine efficacy may vary by population, epidemiological context and history of gonorrhoea. The completed trials in MSM were conducted in populations with very high gonorrhoea acquisition, frequent asymptomatic and extragenital gonorrhoea, and ongoing exposure risk. In contrast, most observational studies have evaluated younger populations with lower incidence, predominantly symptomatic urogenital gonorrhoea and less intensive screening.

Results from ongoing RCTs in women and lower‐risk populations will be critical to defining the role of 4CMenB in gonorrhoea prevention. Alongside this, post‐implementation surveillance in the UK and Galicia may identify effects on transmission or duration of infection not captured by trial endpoints but relevant to population‑level impact. These insights will inform both vaccine policy and the development of next‐generation gonococcal vaccines.

## Author Contributions

All authors contributed to the writing and editing of this manuscript.

## Conflicts of Interest

KLS and AEG were the lead investigators of the GoGoVax clinical trial that received the 4CMenB vaccine from GSK.

## Data Availability

Data sharing is not applicable to this article as no datasets were generated or analysed during the preparation of this commentary.
